# Implementing enhanced extracorporeal membrane oxygenation for CPR (ECPR) in the emergency department

**DOI:** 10.1186/s12245-024-00652-y

**Published:** 2024-06-10

**Authors:** Matthew Oliver, Andrew Coggins, Natalie Kruit, Brian Burns, Brian Plunkett, Steve Morgan, Tim J. Southwood, Richard Totaro, Paul Forrest, Saartje Berendsen Russell, Ruaidhri Carey, Mark Dennis

**Affiliations:** 1 Department of Emergency Medicine, Royal Prince Alfred Hospital, Sydney, Australia; 2 University of Sydney Medical School, Sydney, Australia; 3 Greenlight Institute for Emergency Care, Royal Prince Alfred Hospital, Sydney, Australia; 4 Department of Emergency Medicine, Westmead Hospital, Sydney, Australia; 5 Department of Anaesthesia, Westmead Hospital, Sydney, Australia; 6 Aeromedical Retrieval Services, New South Wales Ambulance, Sydney, Australia; 7 Department of Cardiothoracic Surgery, Royal Prince Alfred Hospital, Sydney, Australia; 8 Department of Intensive Care Services, St Vincent’s Hospital, Sydney, Australia; 9 Department of Intensive Care Services, Royal Prince Alfred Hospital, Sydney, Australia; 10 Department of Anaesthesia, Royal Prince Alfred Hospital, Sydney, Australia; 11 Department of Cardiology, Royal Prince Alfred Hospital, Sydney, Australia

**Keywords:** ECMO, ECPR, Cardiac, Arrest, Resuscitation

## Abstract

Refractory out-of-hospital cardiac arrest (OHCA) has a very poor prognosis, with survival rates at around 10%. Extracorporeal membrane oxygenation (ECMO) for patients in refractory arrest, known as ECPR, aims to provide perfusion to the patient whilst the underlying cause of arrest can be addressed. ECPR use has increased substantially, with varying survival rates to hospital discharge. The best outcomes for ECPR occur when the time from cardiac arrest to implementation of ECPR is minimised. To reduce this time, systems must be in place to identify the correct patient, expedite transfer to hospital, facilitate rapid cannulation and ECMO circuit flows. We describe the process of activation of ECPR, patient selection, and the steps that emergency department clinicians can utilise to facilitate timely cannulation to ensure the best outcomes for patients in refractory cardiac arrest. With these processes in place our survival to hospital discharge for OHCA patients is 35%, with most patients having a good neurological function.

**Supplementary Information:**

The online version contains supplementary material available at 10.1186/s12245-024-00652-y.

## Introduction

**Fig. 1:**
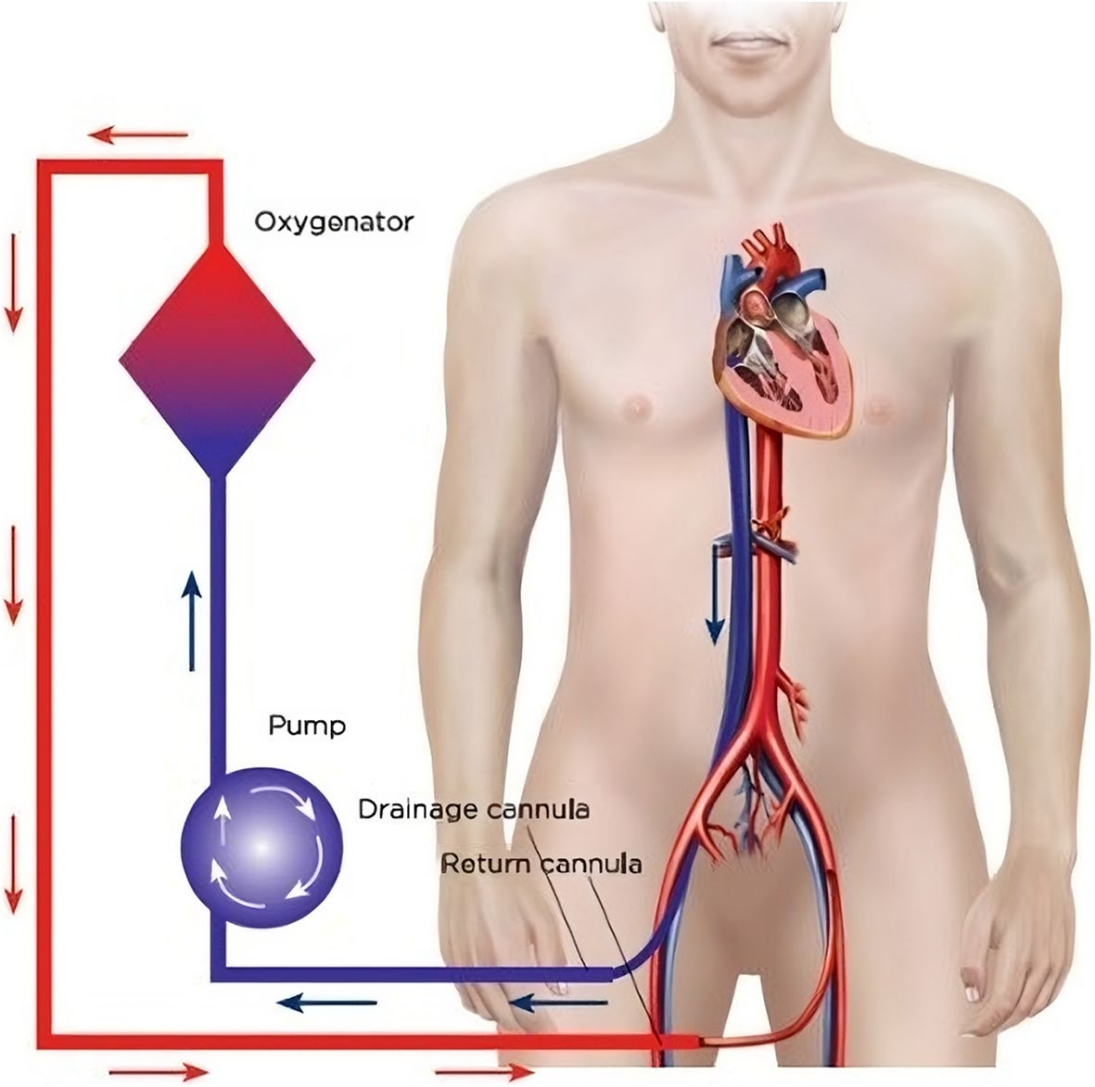
A schematic of an ECPR setup for cardiac arrest. Blood is taken out via the venous ‘drainage’ catheter which enters via the femoral vein with the tip located at the level of the right atrium. Oxygenated blood is pumped back to the patient via an arterial ‘return’ cannula located in the descending aorta. Picture obtained from LearnECMO.com with permission [11]

In an Australian setting, meaningful survival to good neurological outcome following out-of-hospital cardiac arrest (OHCA) is approximately 10% [1–4]. Refractory cardiac arrest (RCA) has a very poor prognosis (< 2% survival) and is often defined as those patients without sustained return of spontaneous circulation (ROSC) following 10–15 min of best-practice resuscitation or after 3 defibrillations for shockable rhythms [5–7]. Extracorporeal membrane oxygenation implemented during cardiopulmonary resuscitation (ECPR) provides end-organ perfusion to patients whilst the underlying cause of arrest can be treated, or recovery occurs (Fig. 1). Despite substantial increase in the use of ECPR, outcomes vary widely. A recent systematic review and meta-analysis of ECPR use for selected OHCA cases demonstrated a survival to hospital discharge of 24% of patients with 18.5% having a favourable neurological outcome.[8] Other systematic reviews and meta-analyses have also demonstrated a survival benefit for cardiac arrest patients receiving ECPR compared to conventional CPR [5, 9, 10].

A key prognostic variable for ECPR is minimising the time from cardiac arrest to the commencement of ECMO flow, a period referred to as the low flow time. Evidence demonstrates that patients who are placed on ECPR beyond 60 min have a very low likelihood of meaningful survival [12–15]. Therefore, prehospital teams and emergency departments (ED) must incorporate systems to facilitate rapid assessment, transport, and ECMO cannulation to achieve the best outcome for carefully selected patients in RCA.

The aim of this brief narrative review is to describe the collective system improvements and implementation strategies that incorporate ECPR into prehospital and ED RCA algorithms in Sydney, Australia.

### Setting

In 2020 the population of greater Sydney in New South Wales (NSW), Australia was just over 5.3 million living in a total area of 12,368.2 Sq km [16]. Currently three main hospitals provide ECPR to greater Sydney, two of which are located in central Sydney. Through shared experience and knowledge, the three tertiary-level hospitals have each established protocols and processes for ECPR within Sydney.

### Our experience

We have previously published our experience of ECPR during the 2CHEER trial and demonstrated a 44% (11/25) survival to hospital discharge with favourable neurological outcome [17]. Since the publication of this data from 2020 to 2023 we have had 51 ECPR cases (Table 1) with 16 survivors to hospital discharge (31%), of these patients 26 were OHCA with 9 survivors (35%). The majority of patients who survived to hospital discharge had good neurological function with the mean CPC score or 1.4 (STD 0.8). This fall in survival rate may reflect real-world experience outside of a clinical trial as well the challenges of managing cardiac arrests during the Covid pandemic.

**Table 1:** Outcome data for ECPR patients from 2020–2023 at the 3 ECPR centres in Sydney

**Patient outcome data—2020–2023**	
Number of ECPR patients—n	51
Number of OHCA patients – n	26
Discharged from hospital alive – n (%)	16 (31)
Discharged from hospital alive OHCA – n (%)	9 (35)
Mean (STD) CPC score at discharge from hospital	1.4 (0.8)

*CPC* Cerebral performance category

### Activation process

A robust and protocol driven prehospital system should facilitate the early activation of key staff and equipment preparation at the receiving hospital in potential ECPR candidates. This requires an awareness that optimising prehospital resuscitation is a critical step and there is a balance between achieving the highest likelihood of ROSC with good prehospital resuscitation whilst also minimising delays to transfer to hospital for ECPR [18]. For patients in RCA meeting criteria for ECPR small delays in transport may be associated with poorer outcomes and so it is critical to minimise the low flow time prior to ECMO initiation [19]. Our prehospital transport times and times to ECMO flows are demonstrated in Table 2.

**Table 2:** Key timepoints of patients with OHCA including prehospital times and times to ECMO flows

Patient data—2020–2023 – OHCA	Total (*n* = 26)
Arrest time to patient contact—mins (SD)	8 (2)
Scene time—mins (SD)	36 (6)
Transport time—mins (SD)	12 (7)
ED arrival to cannulation—mins (SD)	14 (9)
Cannulation to ECMO flows—mins (SD)	15 (7)
ED arrival to ECMO flows—mins (SD)	28 (19)
Arrest time to ECMO flows-mins (SD)	72 (5)
MCPR n (%)	24 (93)

*MCPR* Mechanical-CPR

The standard process for prehospital care in the Sydney system involves the dispatch of an ambulance unit that often comprises of two paramedics with standard ALS skills with the average time of arrival of 8 min [20]. The ALS algorithm is followed with early defibrillation, basic airway management and IV access as well as advanced airway management (supraglottic or intubation) and placement of a MCPR device (LUCAS) [21].

**Fig. 2:**
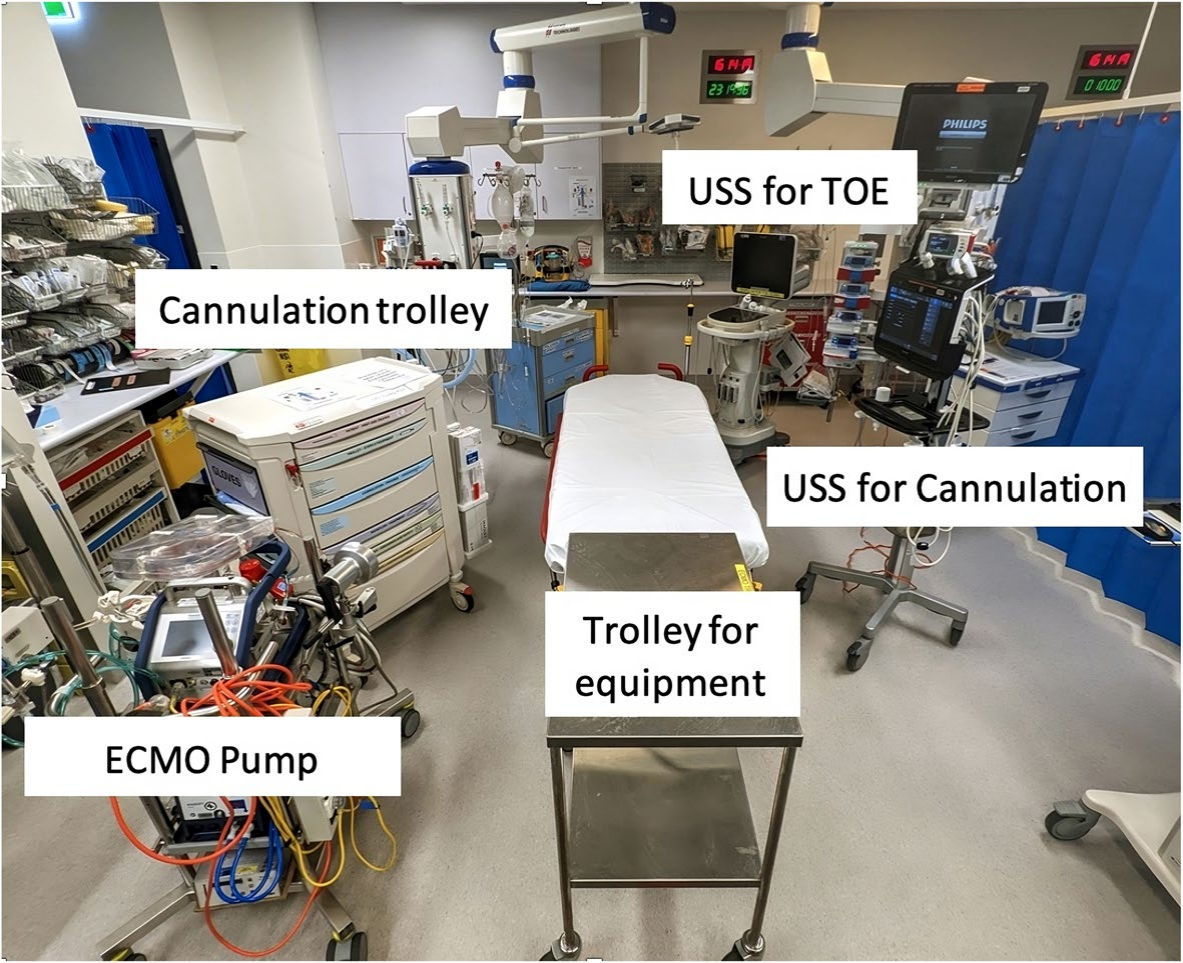
Layout of the emergency department resuscitation bay for ECPR. US = Ultrasound, TOE = Transoesophageal Echocardiography

Once the prehospital notification has been received at the ECPR centre, the relevant team members are activated via a dedicated ECMO/ECPR page, and the resuscitation bay is prepared (Fig. 2). The ECPR team is available at all sites in standard working hours. Occasionally the ECPR team may be activated after working hours, but these are usually on a case-by-case basis and dependent on the availability of the team and the anticipated prehospital transport times.

The criteria to activate the ECPR teams differs slightly between institutions. However, the principles are similar with a balance struck between sensitivity and specificity of the activation criteria. These ECPR activation criteria use weighted factors associated with improved probabilities of survival based on the best available evidence or recommendations [12, 22–26] and include:

- Estimated age 18–70 years.
- Witnessed OHCA.
- Initial rhythm VT/VF or automatic external defibrillator shock delivered or PEA arrest
- Bystander CPR started within < 5 min and ongoing on ambulance arrival with no history suggestive of a prolonged period without CPR

**Fig. 3:**
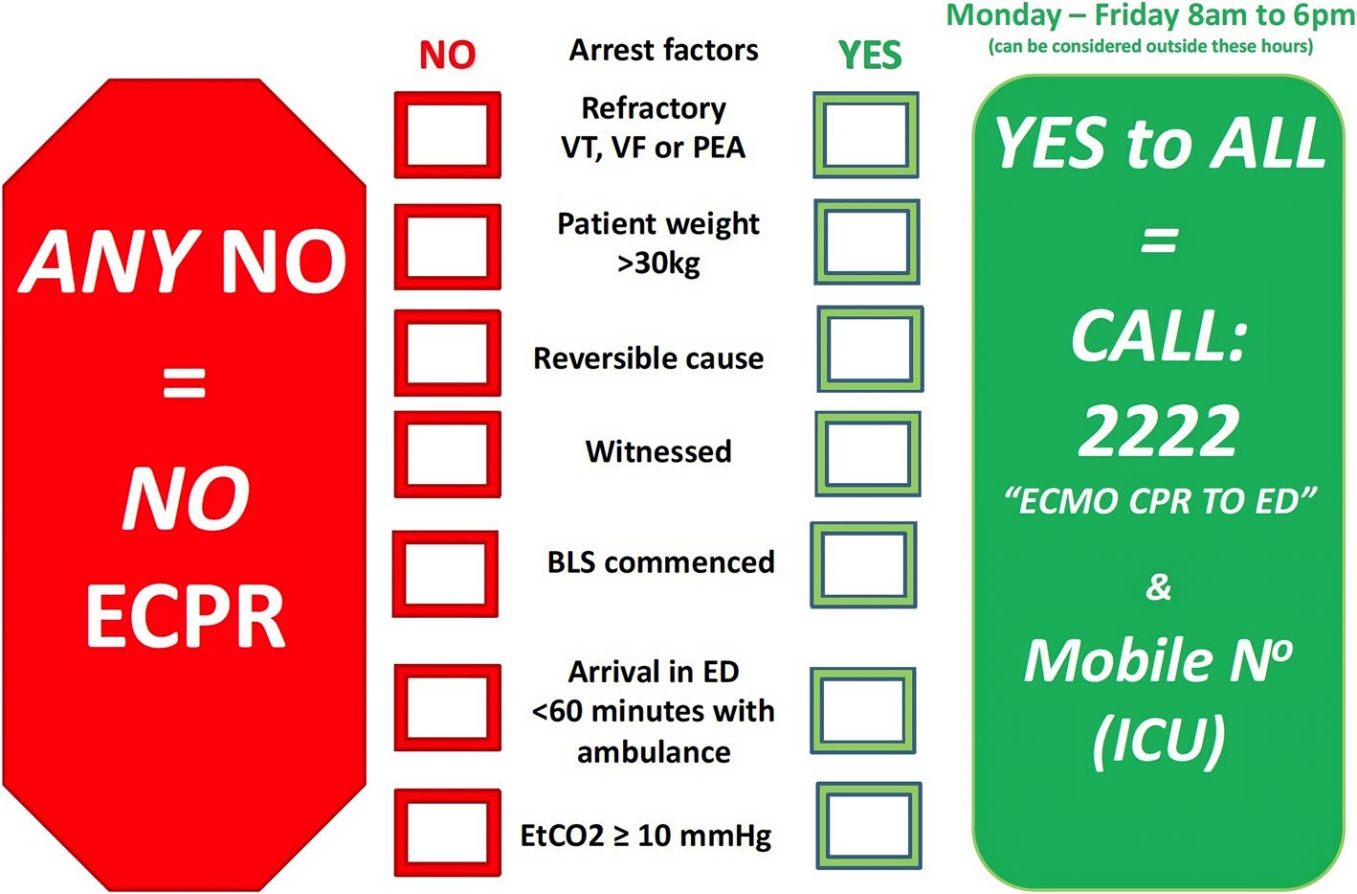
Inclusion and exclusion criteria utilised to activate the ECPR team (RPA hospital)

Figure 3 shows an example of the criteria used for activation within one ECPR centre. The same criteria and activation process are used to mobilise the ECPR team for patients who suffer RCA in the ED.

It is recommended that an institution commencing an ECPR program adheres to strict inclusion/exclusion criteria to avoid including patients who are unlikely to benefit from the intervention and therefore worsen survival rates. Inclusion "creep” is a challenge for many clinicians, especially when managing younger patients. An agreement of predefined criteria from the key stakeholders as well as a local governance structure within the hospital prior to commencement of an ECPR program improves compliance and facilitates efficient management within the ED.

### Roles

**Fig. 4:**
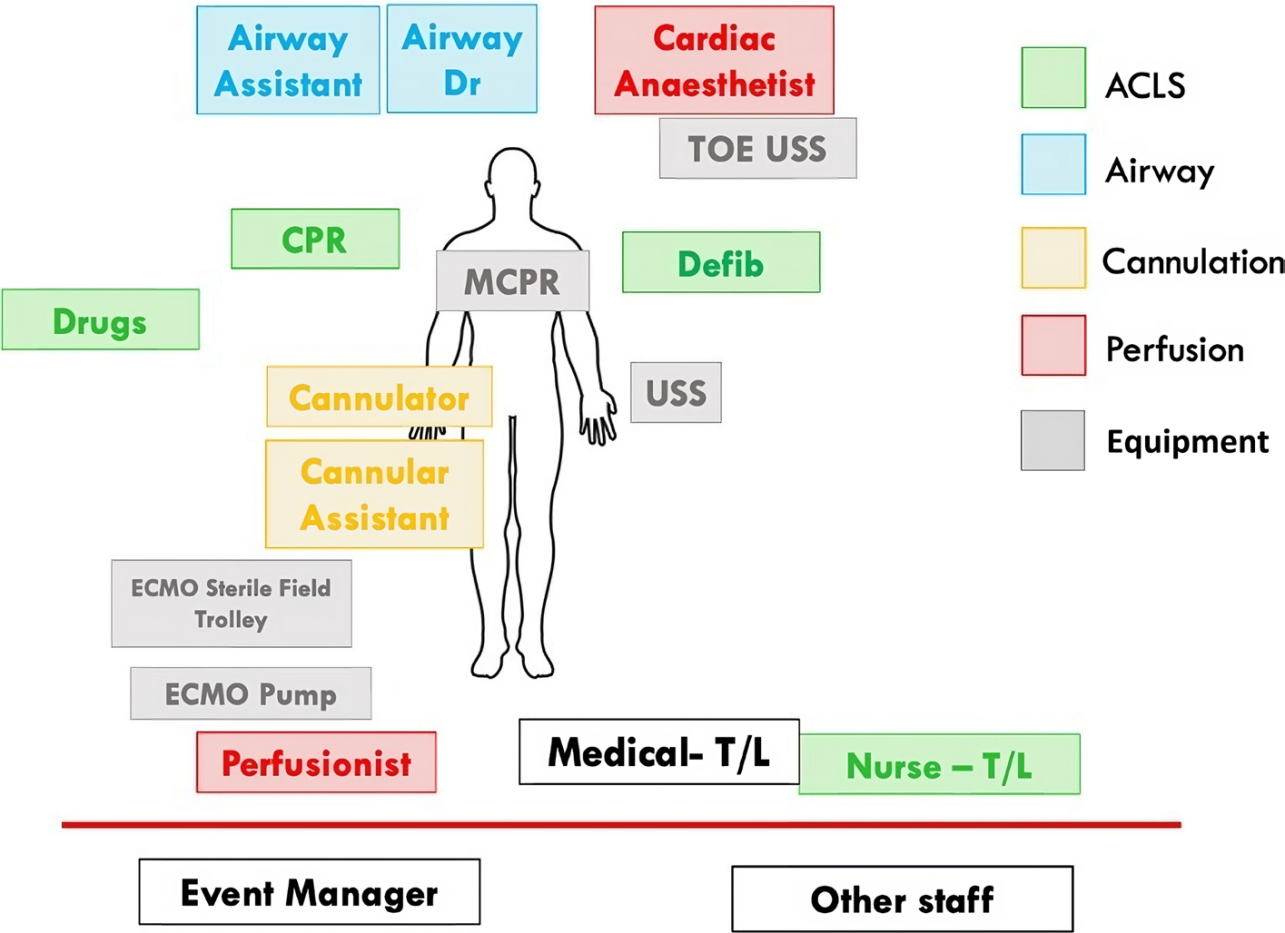
A schematic of staff positions and roles including differentiation into various sub-groups. Any staff members not directly involved in the resuscitation are advised to remain behind a visible red line in the resus bay

It is prudent that the resuscitation team roles are pre-allocated and that key tasks are outlined (Fig. 4 and Appendix 1) to achieve efficient workflow. To maximise the productivity of the members, the team is separated into small sub-teams each with delineated roles and tasks. Further, the use of a nursing team leader to manage operational ALS concerns, and/or a second medical team leader for "event management” (see below) is often pertinent because the cognitive load on a single leader is likely to be overwhelming. Over a series of 7 ECPR calls at one of our sites there was an average peak attendance of 24 staff members (range 16–30) emphasising the challenge of managing both allocated roles and interested staff bystanders.

On arrival to ED the team members should don the appropriate personal protective equipment (PPE) and lanyards that identifies the persons role within the team (Appendix 1). The majority of the ECPR centres utilise a cardiac anaesthetist to perform a transoesophageal echocardiogram (TOE) during the arrest. The TOE becomes essential for: 1. Identifying a potential cause of arrest i.e., pericardial tamponade, visualisation of embolism, aortic dissection 2. Confirming correct wire/cannula placement for ECMO and 3. Ensuring effective left ventricle compression via MCPR [27–29].

The cannulation team are often Intensive Care specialists or Cardiothoracic surgeons and consist of one cannulator plus an assistant positioned to right side of the patient to access the right femoral artery and vein.

A nurse is assigned the role of CPR to ensure that chest compressions are adequate, that the MCPR device is secured in the correct location, and to take over the role of chest compressions if the MCPR device fails.

ECPR cases may require duplication of various roles due to the workload required for various tasks (e.g. medication preparation listed in appendix 2) and attract bystander interest that are in addition to the list active roles (Fig. 4). Therefore, as with any major resuscitation in the ED, crowd control and communication to team members is crucial. Strategies that actively mitigate the latent threat of large numbers of responders and role redundancy are a key consideration in ECPR cases. Having a dedicated team member(s) to manage crowd control, noise levels and perform regular summaries may be efficacious but have not been studied in clinical studies.

### Resuscitation

**Fig. 5:**
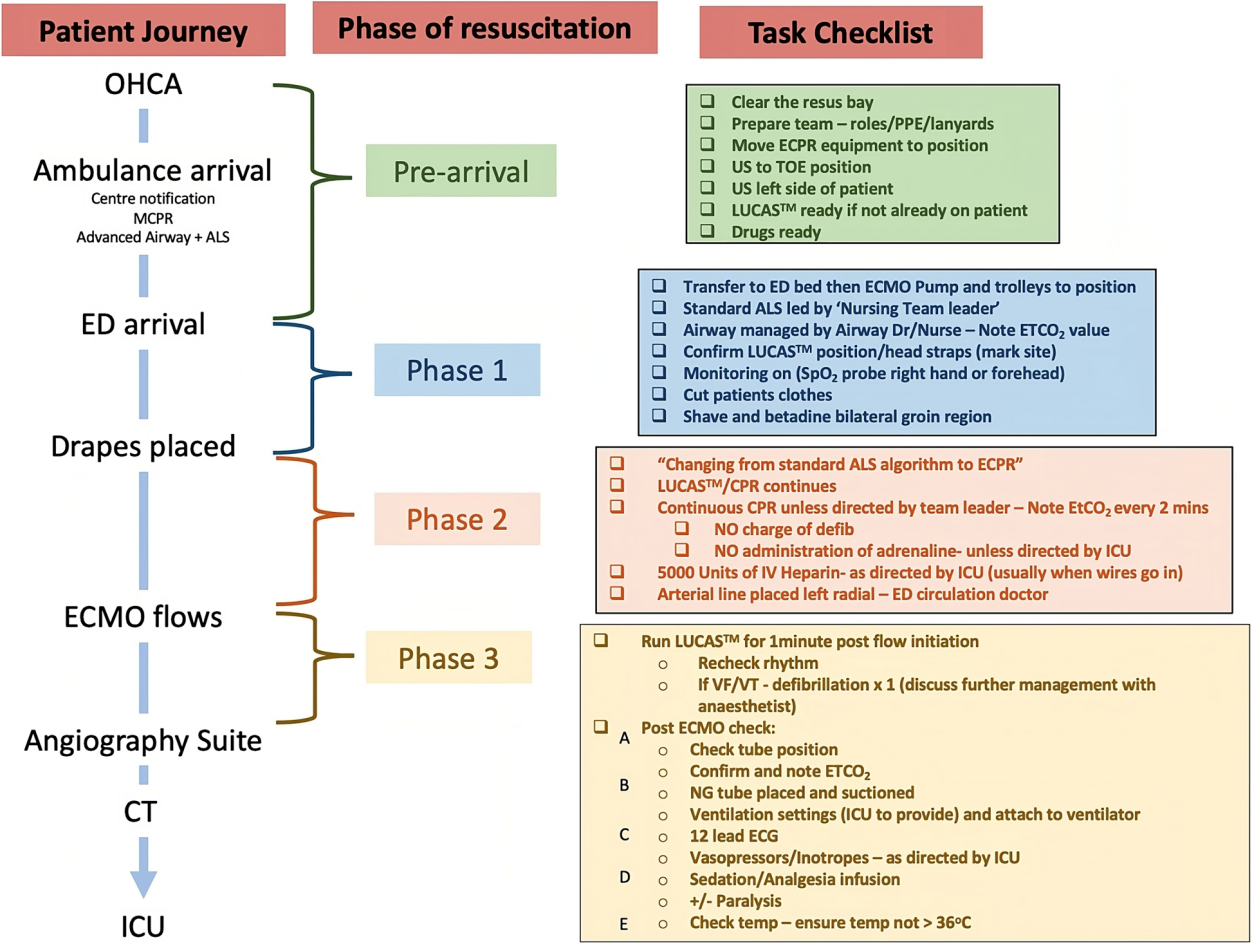
A flowchart of the ECPR patient journey and task priorities for each phase of the resuscitation. Phase 1 = Arrival and decision. Phase 2 = Proceed with cannulation. Phase 3 = Preparation for disposition. MCPR = Mechanical CPR; CT = Computerised tomography; ICU = Intensive care unit; US = Ultrasound; TOE = Transoesophageal Echocardiogram; ETCO_2_ = End-tidal Carbon Dioxide; SpO_2_ = Oxygen saturations; NG = Nasogastric; CXR = Chest x-ray

The resuscitation of ECPR patients is divided into four main phases (Fig. 5). These phases of resuscitation have been adapted from the 'Learn-ECMO’ team and allows for team members to smoothly transition through a complicated resuscitation [11]. Dividing this complex and stressful resuscitation into different phases with key task priorities ensures that all staff members are focused on manageable goals that can be achieved in a timely manner [30]. The team leader utilises a checklist with these tasks for each phase to reduce cognitive burden during the resuscitation.

### Pre-arrival phase

Once the notification has been received at the ECPR centre and the team is activated, the priority is to ensure the staff, drugs (Appendix 2) and equipment are ready to receive the patient. All members of the team don standard resuscitation PPE including eye protection, gowns, and gloves. The cannulation team will prepare for the procedure using standard surgical aseptic technique. The resuscitation bay needs to be large enough (suggested a minimum 25m^2^) to allow for the ECPR equipment and 360-degree access to the patient by nursing and medical staff. Non-essential equipment should be removed and equipment that is essential for cannulation moved into the correct positions (Fig. 2). One head of bed US is used to perform TOE and one or two additional adjacent US machines are to assist with vessel cannulation.

#### Phase 1 Arrival and decision

Phase 1 of the resuscitation begins when the patient arrives in ED and ends when drapes are placed. The patient is rapidly transferred to a resuscitation bed and an abbreviated handover that focuses on the ECPR inclusion/exclusion criteria by the paramedics. The ED nursing team leader will continue the cardiac arrest algorithm as per standard ALS recommendations. During this time the ED team leader and ECMO cannulators perform a rapid assessment of whether the patient is suitable using the criteria mentioned previously. Once the patient is deemed appropriate then a list of tasks is utilised by the ED team leader to prepare the patient for cannulation. If not already performed in the field, then an endotracheal tube is placed and secured by the most senior ED doctor, facilitating the ability to perform a TOE.

#### Phase 2 – Proceed with cannulation

The commencement of Phase 2 is indicated once the sterile drapes are placed for cannulation. The medical team leader must notify staff that the team is no longer performing standard ALS management as defibrillation and adrenaline dosing are paused in preference for successful cannulation. MCPR is performed continuously with only very brief interruptions if cannulation is challenging. Ideally an arterial line is placed by the ED or ICU 'circulation’ doctor. Intravenous heparin 5000units is given as the guidewires are placed.

#### Phase 3 – Preparation for disposition

Once ECMO flows are established a myriad of challenges and complexities are possible that are outside the scope of this article or standard practice for the emergency physician. Some of these acute challenges include haemodynamic instability, coagulation management and circuit complications. A list of key priorities to mitigate these challenges are described in Fig. 5. In our experience we have found that once adequate ECMO flow is established then patients frequently revert to malignant reperfusion rhythms including VF. Approximately one minute of CPR helps to decompress the distended right ventricle that frequently occurs during cardiac arrest followed by defibrillation if in a shockable rhythm.

Often whilst in ED awaiting transfer to either the cardiac angiography suite, CT, or ICU a distal perfusion cannula is placed in the superficial femoral artery ipsilateral to the arterial return cannula. The distal perfusion cannula is again placed under US guidance and ensures adequate antegrade perfusion to the lower leg as flow distal to the common femoral artery is limited or absent due to the size of the arterial return cannula.

A nasogastric tube is placed in ED so that antiplatelet medications (Aspirin and Clopidogrel or Ticagrelor) can be commenced for patients with a suspected coronary artery cause. Further anti-coagulation management is completed in ICU and under the direction of the perfusion or ICU teams and may be directed by activated clotting time (ACT), APTT/anti-Xa and/or visco-elastic testing. Once the patient is ready the medical team leader hands over care of the patient to the cardiac anaesthetist for transfer.

### Post- ED care

Activation of the cardiac angiography suite occurs routinely on the notification of a potential ECPR patient arriving with OHCA. Immediate coronary angiography is performed after establishment of ECMO flow. However, if during the resuscitation the ED team leader in communication with the resuscitation team feels that another cause is more likely, then the angiography can be deferred. Whilst controversy remains regarding the utility of empiric angiography for patients post cardiac arrest of unknown cause or without ST Elevation, many of the recent trials did not include haemodynamically unstable patients and patients without ROSC or ECPR [31–33]. Following cardiac arrest the presence of acute coronary lesions in patients with non – ST elevation cardiac arrests vary by 40–70%, however, this prevalence is increased in refractory cardiac arrest patients [31, 32, 34]. In our experience, 61% (*n *= 20) of ECPR patients who had a coronary angiogram (*n* = 33) required stenting of a coronary lesion indicating that there are a significant proportion of ECPR patients that may benefit from cardiac angiography.

Prior to ICU admission a computerised tomography (CT) of the head, and chest to knees is performed to identify any evidence of intracranial pathology (e.g. intracranial haemorrhage) or sequelae of chest compressions (e.g. traumatic pneumothorax, rib fracture, solid organ trauma), and cannulation injuries and configuration. In approximately 40% of CT pan scans a pathology will be identified that will change the management of patients [35].

## Discussion

ECPR is rapidly evolving into a recognised practice for patients in cardiac arrest in the emergency department. We report a pragmatic approach to how we perform ECPR within our centres, our experience and lessons learnt over the years within our institutions, and differences from the recent modified Delphi technique that identified broader best practices in relation to ECPR [36]. Importantly we divide the resuscitation of ECPR patients into several key steps with delineated phases (e.g. Sterile drapes being placed on the patient indicating phase 2) that allow for the medical team leader to guide staff through what can be perceived as a complicated resuscitation. Each phase has important tasks that can be presented in the form of a checklist. Another key principle of our practice is the efficient integration of multiple teams ensuring a streamlined process of patient care from the prehospital environment through to the ICU.

To date the evidence of effectiveness for ECPR is limited with only four randomised clinical trials [19, 37–39]. Three of these studies showed no difference in outcomes for patients receiving ECPR compared to those receiving conventional CPR (CCPR) [37–39]. The latest study by Suverein et al. randomised 160 patients aged 18-70yrs old with witnessed refractory OHCA, with bystander CPR and shockable rhythms to either ECPR or CCPR.[39] At 30 days, 20% of the ECPR group were alive with a favourable neurological outcome compared to 14% in the CCPR group (odds ratio 1.4; 95% CI 0.5 to 3.5; *P* = 0.52). Despite relatively short prehospital times in the ECPR group (mean time of 36 ± 12 min) and the CCPR group (mean time 38 ± 11 min) the average time to ECMO flows for the ECPR group was over 60 min for the majority of patients (mean time 74 min IQR 63–87). This data indicates that the time to perform cannulation and achieve flows is on average 38 min, possibly accounting for the poor outcomes in the ECPR group and further emphasising the need for end-to end cardiac arrest system, optimisation, and training.

The only study to demonstrate a benefit of ECPR by Yannopoulos et al., 30 patients were randomised to ECPR or CCPR with only one patient (7%) surviving in the CCPR group compared to 6 in the ECPR group (43%).[19] The trial was stopped at a planned interim analysis which demonstrated superiority of ECPR. The mean time from 911 call to ECMO initiation was 59 min in this study. Although this study group had a more selective inclusion criteria compared to the other trials (including the use of biochemical markers) it highlights the importance of strict patient selection criteria to maximise the chance of good outcomes for patients [40]. Future studies may be able to provide more evidence to novel prognostic indicators to aid clinicians when considering ECPR activation.

A recent meta-analysis by Low et al. demonstrates a mortality benefit of ECPR compared to CCPR for OHCA patients (OR 0.62, 95% CI 0.45–0.84) that included favourable short term 30-day neurological function [41]. Our experience has demonstrated a 35% survival rate for ECPR patients with OHCA with the majority having a good neurological function (mean CPC 1.4). This demonstrates an overall improvement on 10% survival for OHCA patients receiving CCPR. We recognise that our mean time of 72 min from the time of arrest to ECMO flows is longer than the recommended 60 min and therefore we are in the process of conducting a randomised control trial investigating patient outcomes for expedited transfers from the scene versus standard care for OHCA patients [42].

## Conclusion

To achieve the best outcome for selected patients with refractory OHCA changes to prehospital and hospital systems need to be adapted if an ECPR program is to be utilised. These changes include having strict activation criteria, role allocation and a clear resuscitation plan that will maximise the performance of the team members and minimise the time to achieve perfusion via ECMO. We provide our processes to achieve the best possible outcomes.

### Supplementary Information

Supplementary Material 1.

## Data Availability

Not applicable.

## Abbreviations

ECMO: Extracorporeal membrane oxygenation
ECPR: ECMO cardiopulmonary resuscitation
OHCA: Out-of-hospital cardiac arrest
ROSC: Return of spontaneous circulation
RCA: Refractory cardiac arrest
ED: Emergency Department
ALS: Advanced life support
MCPR: Mechanical CPR
VF: Ventricular fibrillation
VT: Ventricular tachycardia
PEA: Pulseless electrical activity
TOE: Transoesophageal echocardiogram
US: Ultrasound
CT: Computerised tomography
