# Supplementary material for: Implementing enhanced extracorporeal membrane oxygenation for CPR (ECPR) in the emergency department
Source: Int J Emerg Med. 2024 Jun 10;17:71. doi: 10.1186/s12245-024-00652-y (PMC11163769; doi:10.1186/s12245-024-00652-y)
Supplement: Supplementary file 1 — Supplementary Material 1. [file 12245_2024_652_MOESM1_ESM.docx]

**Appendix 1- Roles**


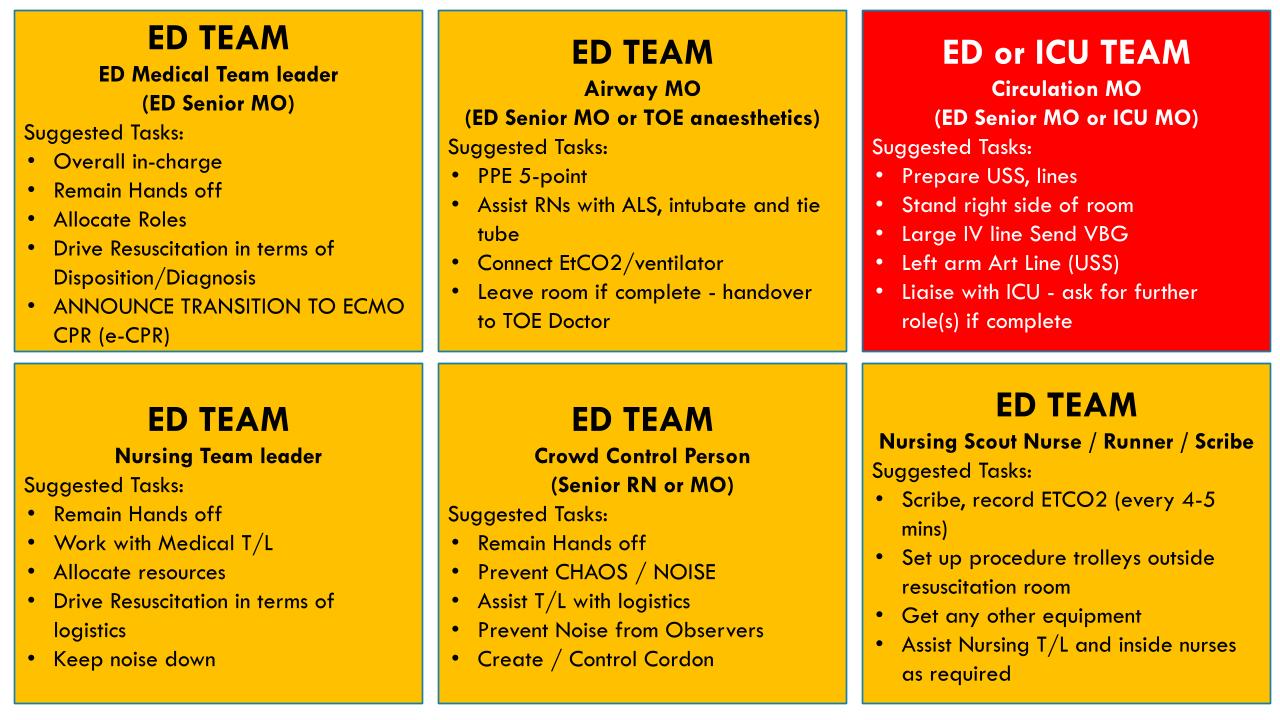


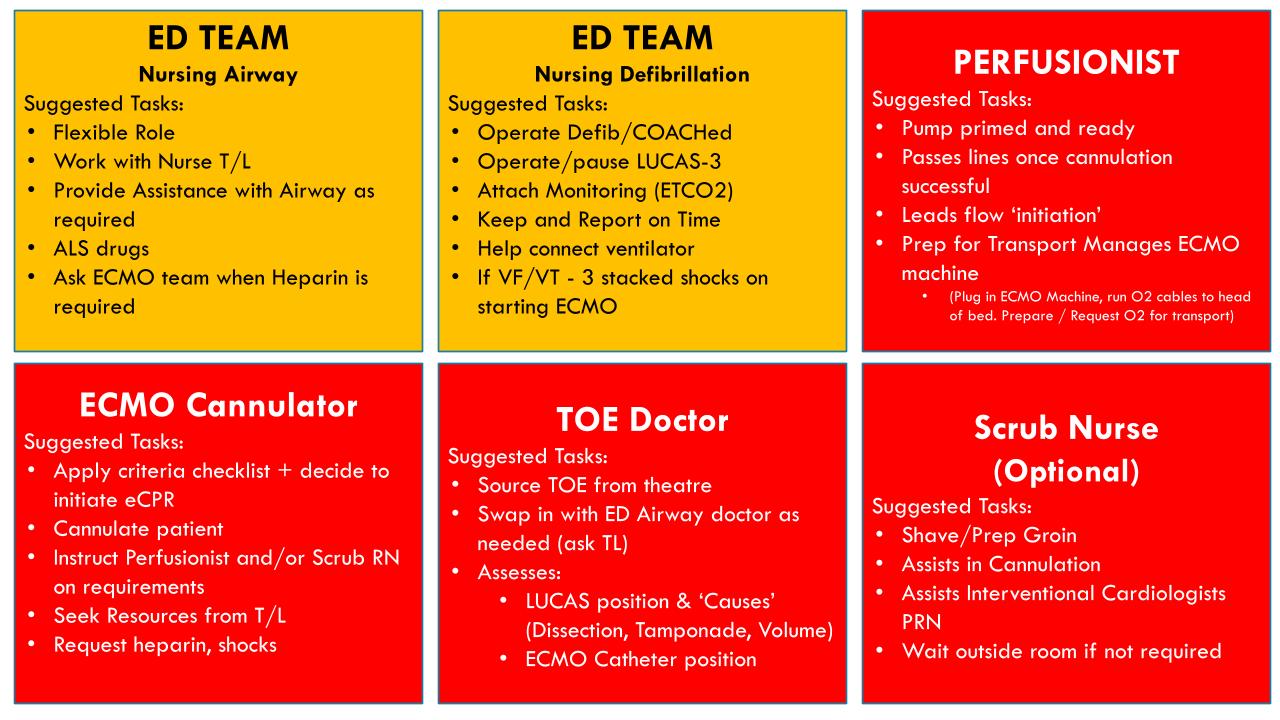


**Appendix 2- Drugs**

ALS Drugs:

- **Adrenaline** 1mg in 10 mLs neat x 3
- **Amiodarone** 300mg in 20mls 50% dextrose

Vasopressor:

- **Metaraminol** 10mg in 20mLs Normal Saline

Anticoagulation: **Heparin** 5000iu neat

Sedation/Paralysis

- **Morphine** 50mg in 50mls N/Saline
- **Midazolam** 50 mg in 50mLs N/Saline
- **Rocuronium** 50mg neat

Antibiotics: **Cephazolin 2g**
